# Gender impact on quality of life in colorectal cancer survivors

**DOI:** 10.2478/raon-2025-0023

**Published:** 2025-04-11

**Authors:** Aleksandra Grbic, Majda Causevic, Sara Brodaric, Mojca Birk, Irena Oblak

**Affiliations:** Division of Supportive Treatment and Joint Health Activities, Institute of Oncology Ljubljana, Ljubljana, Slovenia; Information Technology Department, Institute of Oncology Ljubljana, Ljubljana, Slovenia; Epidemiology and Cancer Registry, Institute of Oncology Ljubljana, Ljubljana, Slovenia; Department of Radiotherapy, Institute of Oncology Ljubljana, Ljubljana, Slovenia; Faculty of Health Sciences, University of Primorska, Slovenia; Faculty of Medicine, University of Ljubljana, Ljubljana, Slovenia

**Keywords:** gender, quality of life, late effects, colorectal cancer

## Abstract

**Background:**

The aim of the study was to evaluate gender-specific differences in the quality of life (QoL) and late effects among colorectal cancer patients during the first two years after treatment, to inform and improve long-term follow-up care and clinical management strategies.

**Patients and methods:**

A total of 239 colorectal cancer patients were included, 56% males and 44% females, mostly in the age range 60–69 years. They were treated at the Institute of Oncology Ljubljana, during the time period from 1^st^ September 2023 to 1^st^ May 2024. In addition to demographic data, we included clinical data on disease and outcomes collected using the standardized quality of life questionnaires of European Organization for Research and Treatment of Cancer (EORTC) named EORTC QLQ-30 and EORTC QLQ-CR29 for colorectal cancer, respectively.

**Results:**

Females were more likely to experience emotional problems (p = 0.002), higher levels of fatigue (p < 0.001), insomnia (p = 0.015), nausea and vomiting (p = 0.007), which may also be associated with poorer appetite in females. Males reported better body image than female (p = 0.047), lower levels of anxiety (p = 0.029), less frequently reported perceived weight loss or gain (p = 0.010). Male reported more stool frequency (p = 0.045), and also had more sever dysuria compared to female (p = 0.008).

**Conclusions:**

The results provide the opportunity to improve the clinical management of long-term follow-up and care planning, taking into consideration the gender-specific needs of colorectal cancer survivors.

## Introduction

According to global cancer statistics (GLOBOCAN 2020), colorectal cancer (CRC) ranks among the three most prevalent cancers globally in both incidence and mortality.^1,2^ The estimated five-year relative survival rate stands at approximately 67%.^3^

Data from the Cancer Registry of the Republic of Slovenia at the Institute of Oncology Ljubljana (OIL), indicate that in 2020, Slovenia recorder 1304 new cases, with 681 associated deaths. CRC is the fourth most common malignancy in both genders, with highest incidence observed in individuals over 75 years of age. Over the recent decades, CRC survival rates have markedly improved due to the implementation of SVIT screening programme (leading to an annual decline in crude incidence rates of 1.6%)^4^, early detection and advances in treatment.

The one-year survival rate in Slovenia (2016–2020) for men patients diagnosed with colon cancer was 79.6%, while for rectal and rectosigmoid junction cancer, it was 84.1%. In women, these rates were 80.0% and 80.2%, respectively. The five-year survival rate for colon cancer in men was 63.0%, compared to 63.2% in women, while survival rates for rectal and rectosigmoid junction cancer were 63.4% in men and 59.3% in women.^4^

Health-related quality of life (HRQoL) is defined as a multidimensional assessment^5^, focusing on the impact of disease and its treatment on a patient’s subjective well-being.^6^ Research has highlighted the importance of HRQoL in cancer patients, emphasizing its role in physical, psychosocial and financial burdens, all directly impacting patient outcomes.^7^

Ultimately, overall quality of life is a critical determinant of long-term survival and recovery, influencing the daily functioning and emotional well-being.^7,8^ EORTC QLQ-C30 (C30) and EORTC QLQ-CR29 (CR29) questionnaires, developed by the European Organization for Research and Treatment of Cancer (EORTC), are widely utilized to assess patient-reported outcome measures (PROMs) in CRC patients from diagnosis through treatment and follow-up. The C30 questionnaire evaluates the general health-related quality of life in cancer patients across various cancer types, while CR29 module supplements this assessment with CRC-specific concerns.^9^

Approximately half of CRC survivors experience late treatment effects^10^, which may manifest months or years post-treatment, encompassing both physical and psychosocial complications. Late systemic treatment side effects include peripheral neuropathy, fatigue, cognitive impairment, while post-operative complications may involve stoma-related issues, urogenital and sexual dysfunction, diarrhea, bloating, flatulence, incisional hernia and increased risk of bowel obstruction. Radiotherapy related late side effects may include urogenital and sexual dysfunction, bloating, diarrhea, incontinence, abdominal pain, sore skin, infertility, increased risk of fractures and bowel obstruction.^11^

These persistent physical symptoms contribute to stress, feelings of insecurity and psychological distress in CRC survivors. During follow-up, CRC survivors often experience anxiety and fear of a recurrence, particularly those with progressive disease. The psychological well-being is frequently impacted by altered body image due to surgery, weight loss and the presence of stoma.^12^ Notably, patients with a stoma report significantly higher and more sustained distress compared to those without.^13^ Social functioning is also frequently impaired, with studies indicating that bowel dysfunction, stoma-related problems and changed body image concerns contribute to embarrassment, anxiety and withdrawal from social interactions.^12,13^ Racial and ethnic disparities also influence quality of life (QoL) outcomes in CRC patients. A study assessing QoL in 1.132 CRC patients using SF-12 physical (PCS) and mental composite summary (MCS) scores, analyzed sociodemographic associations and survival differences, found that never-married Hispanics had higher odds of poor PCS (P = 0.028). College education appeared to mitigate the risk of poor PCS for Hispanics and White patients but not Black patients. Gender differences in MCS scores were associated with worse survival outcomes, with the most pronounced impact observed in White patients. Furthermore, poor PCS/MCS were associated with worse survival outcomes, with the most pronounced impact observed in White patients, whereas Black patients with poor HRQoL had significantly worse outcomes.^14^

Study 2.492 analyzed the quality of life of CRC survivors. Non-Hispanic blacks (p = 0.045) and Hispanics (p < 0.001) reported poorer QoL compared to non-Hispanic whites. Among the most important risk factors for lower QoL in all groups were unemployment or retirement and low income. Other contributing factors included marital status, rural residence, and low educational attainment, with the strongest interaction observed between Hispanics and education (p = 0.045).^15^ Further research shows that there are racial and ethnic differences in HRQoL in older adults with colorectal cancer. Prior to cancer diagnosis, patients of Asian/Pacific Islander descent had better physical HRQoL than patients of Black/African descent, while White and Black/African patients had better mental HRQoL than Hispanic patients. After diagnosis, patients of Asian/Pacific Islander descent had better mental HRQoL than Hispanic patients. For all groups, cancer diagnosis appeared to have a negative impact on overall HRQoL.^16^

A study that investigated gender-specific differences found that women experience more side effects from treatment. Compared to men, they more frequently reported poorer physical function, nausea and pain. Women are not only more susceptible to physical but also psychological stressors.^6^ Surgical treatment has a negative impact on the sex lives of CRC survivors, especially in younger patients and in men. Women are more likely to have a poorer QoL, despite having higher sexual functioning scores than men.^3^ One study found that women who underwent abdominoperineal resection of rectal cancer were less sexually active and less likely to experience arousal or orgasm than women who had an anterior resection. In men, one study found that total mesorectal surgery impaired erection (80%) and ejaculation (82%), while another found less impact on erection and ejaculation.^17^

A study by Laghousi *et al*. showed that women with CRC had poorer physical (p = 0.001) and social functioning scores (p = 0.038) than men. In addition, women on average suffered more pain and fatigue.^18^ Studies examining gender differences in the QoL of cancer patients often find that women report greater psychological distress, stress, anxiety and depression than men. However, some studies suggest that women are also more willing to report physical and emotional changes, which may influence their lower QoL scores. Women’s more open approach to reporting their problems, as well as their different social roles and social pressures, could therefore be a reason for the significant gender differences in QoL in colorectal cancer patients.^6,18^

This may contribute to them being more willing to report problems than men, who are often brought up to suppress the expression of their feelings and problems. Women are more likely to seek support from friends, family or healthcare professionals, which encourages them to be more open about their concerns. Increased sensitivity to physical and psychological symptoms and seeking help to cope with these problems can lead to a poorer QoL being reported more accurately. Understanding gender differences provides an opportunity to personalize healthcare services^6^, better understand QoL and prognostic factors, and plan appropriate interventions.^19^ The aim of the study was to assess gender differences in quality of life and late effects in colorectal cancer survivors in the first two years after treatment at IOL to inform and improve long-term follow-up and clinical management strategies.

## Patients and methods

### Patients

The study was approved by the OIL Expert Council on 29/08/2023, the Ethics Committee of the OIL ERIDEK-0029/2023, the Commission for the peer review of protocols and clinical trials at the OIL ERID-KSOPKR-0021/2023 and the Commission of the Republic of Slovenia for Medical Ethics of the CME of the Republic of Slovenia (approval number: 0120-192/2023/6), within the framework of the Ph.D. thesis entitled Quality of life in colorectal cancer survivors. The study was conducted in accordance with the ethical standards defined by the Declaration of Helsinki and the Good Clinical Practice guidelines.

The sample for this study consisted of CRC patients with a diagnosis coded as C18-C20 (colon, rectal and rectosigmoid junction) according to the ICD-10 classification, limited to stage I-III disease (no distant metastases). Patients were eligible if they were up to 24-month post-completion of specific oncological treatment and were being followed up by oncologists in the gastroenterology outpatient department of the OIL. The inclusion period spanned from September 1^st^ 2023 to May 1^st^ 2024 and each patient was included only once during this timeframe, regardless of the number of follow-up visits they attended. During this period more patients were identified as eligible for inclusion in the study, but 239 of them completed the questionnaire. The sample included patients diagnosed through the national SVIT colorectal cancer screening program, as well as those diagnosed at the OIL or other healthcare institutions across Slovenia. However, precise data on the origin of diagnosis (*i.e*., specific healthcare facility) were not available. This cohort represents a focused group of CRC patients undergoing standardized followup care within a defined clinical setting and timeframe. Additionally, the patients included in the study were treated at the OIL with curative intent, through surgery, radiotherapy, systemic therapy, or a combination of these treatments, in accordance with national guidelines.

### Questionnaires

The study used a quantitative research method. Patients received the questionnaire only once during a follow-up period of up to 24 months after completion of treatment in the OIL gastroenterology outpatient department. If a patient returned for an outpatient follow-up during this period, they were not re-enrolled in the study. The data were extracted from the OIL information system by checking the patients who were scheduled for follow-up visits to the gastroenterology outpatient department after completing their treatment. The investigator informed the nurse which patients were eligible for the study on the day the outpatient department was in operation. The nurse gave the eligible patient a questionnaire with the patient’s identification number on it. The investigator assigned an ID number to the patient, which was then entered into the investigator’s database.

Care was taken to ensure that patients received only one questionnaire to complete during the follow-up period of up to two years after completion of treatment. Each questionnaire was provided with the patient’s identification number, which was linked to the investigator’s database. Patients’ names were not used directly, but the use of an ID number allowed the data to be traced back to the individual. The questionnaire included demographic data such as gender, age, education and marital status. The standardized C30 questionnaires on quality of life and the additional module of the CR29 questionnaire were used. The C30 includes five functional scales (physical functioning, role functioning, emotional functioning, cognitive functioning, social functioning), three symptom scales (fatigue, nausea and vomiting, pain), a global health status and quality of life scale, and six individual items (dyspnea, insomnia, appetite loss, constipation, diarrhea, financial problems). The CR29 includes 4 multiple-item scales and 19 single-item scales assessing a range of symptoms and problems typical for CRC patients. The scales of symptoms and problems include: urinary frequency, urinary incontinence, dysuria, abdominal pain, buttock pain, bloating, blood and mucus in stool, dry mouth, hair loss, taste change, flatulence, faecal incontinence, sore skin, stool frequency, stoma embarrassment, stoma care problems, impotence, dyspareunia. Functional scales include: anxiety, body weight, body image, sexual interest in men and women. All scales and single item measurements have a score range from 0 to 100. A high score on the functional scale and functional single items indicates a high level of functioning, while a high score on the symptom scale and symptom single items indicates more severe symptoms or problems.^9^

### Statistical analysis

The questionnaires were administered to patients who fulfilled the inclusion criteria of the study by a nurse in gastrointestinal cancer outpatient department at OIL. The collected data were entered into our survey database using IBM SPSS version 29.0. The following statistical methods were used: descriptive statistics (frequency, minimum, maximum, mean, standard deviation), and due to nonnormal distribution of the data (Shapiro-Wilk normality test, p < 0.05) and Mann-Whitney U-test. The level of statistical significance considered is 0.05.

## Results

The study included 239 CRC patients up to 24 months after completion of oncological treatment. Among them, 134 (56.1%) were males and 105 (43.9%) females. Most of the respondents were between 60 and 69 years old (range 30-89+). For both genders, secondary education predominated with 57.7%. 70.2% of the participants were married or living in a common-law relationship. The primary localization of the disease was the rectum (C20) in 52.3% and the colon (C18) or rectosigmoid junction (C19) in 47.7%. The numbers for colon (C18) and rectosigmoid junction (C19) are grouped together or often mentioned together due to their anatomical proximity and their shared characteristics in terms of both cancer risk and treatment approaches.

The disease stage was assessed as stage I in 17.2%, stage II in 22.2% and stage III in 60.7%. The low number of patients with stage I (17.2%) in our study probably reflects the characteristics of the cohort, which was mainly composed of patients who had completed treatment. We included patients who had been treated at the OIL and were undergoing follow-up. In this case, it was expected that a higher proportion of patients would be in stages II and III. The OIL, as a tertiary facility, is more focused on advanced cancer cases that require more complex treatment, while early stages can be treated at other hospitals or healthcare facilities. The general characteristics of CRC patients and gender differences are shown in Table 1.

**TABLE 1.** Socio-demographic and other characteristics of colorectal cancer patients, grouped by gender (n = 239)

Variables	Total number (%)	Male (%)	Female (%)
**Gender**			
Male	134 (56.1)		
Female	105 (43.9)		
**Age**			
30–39	3 (1.3)	1 (0.7)	2 (1.9)
40–49	22 (9.2)	7 (5.2)	15 (14.3)
50–59	46 (19.2)	27 (20.1)	19 (18.1)
60–69	68 (28.5)	49 (36.6)	19 (18.1)
70–79	61 (25.5)	28 (20.9)	33 (31.4)
80–89+	39 (16.3)	22 (16.4)	17 (16.2)
**Education**			
Unfinished primary school	8 (3.4)	4 (3.0)	4 (3.8)
Primary education	43 (18)	16 (11.9)	27 (25.7)
Secondary education	138 (57.8)	86 (64.2)	52 (49.5)
Higher education / university degree	43 (18)	25 (18.7)	18 (17.1)
Master’s degree / Ph.D.	7 (3)	3 (2.2)	4 (3.8)
**Marital status**			
Divorced	11 (4.7)	8 (6.0)	3 (2.9)
Married or common-law	167 (70.2)	101 (75.9)	66 (62.9)
Single	22 (9.2)	15 (11.3)	7 (6.7)
Widowed	38 (16)	9 (6.8)	29 (27.6)
**Primary location**			
Colon (C18) or rectosigmoid junction (C19)	114 (47.7)	54 (40.3)	60 (57.1)
Rectum (C20)	125 (52.3)	80 (59.7)	45 (42.9)
**TNM staging**			
Stage I	41 (17.2)	21 (15.7)	20 (19.0)
Stage II	53 (22.2)	21 (15.7)	32 (30.5)
Stage III	145 (60.7)	92 (68.7)	53 (50.5)
**Treatment**			
Surgical treatment	92 (38.5)	40 (29.9)	52 (49.5)
Combination of radiation and surgery	21 (8.9)	13 (9.7)	8 (7.6)
Combination of radiation, systemic and surgical treatment	96 (40.2)	68 (50.8)	28 (26.7)
Combination of systemic and surgical treatment	30 (12.6)	13 (9.7)	17 (16.2)

### EORTC QLQ C30 scoring scale

**FIGURE 1.**
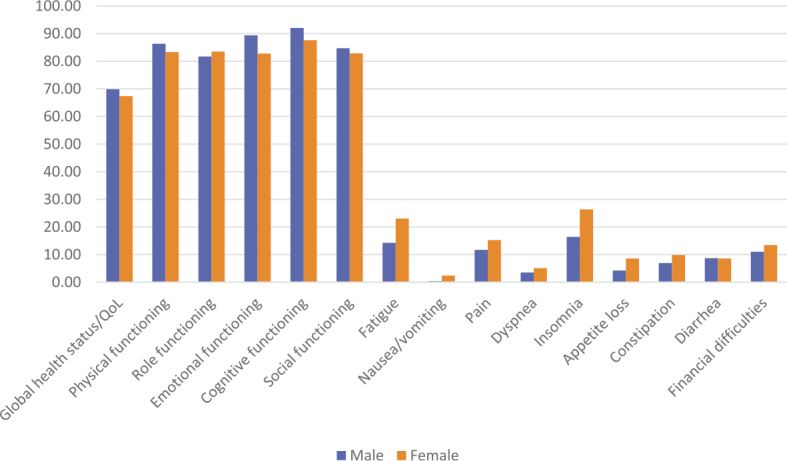
Mean scores between males and females in the standardized quality of life questionnaires of European Organization for Research and Treatment of Cancer (EORTC) named EORTC QLQ-30.

Figure 1 shows the mean scores of the functional scales and symptoms/other items in the C30 questionnaire in CRC patients by gender. Males reported better overall health and domains of functioning, particularly emotional and cognitive functioning, while females experienced greater symptom burden such as fatigue, pain, insomnia, and loss of appetite. The results of the Mann-Whitney U-tests for the functioning scales in Table 2 show that emotional functioning is statistically significantly worse in females compared to males (p = 0.002). Females also suffer more frequently from fatigue (p < 0.001), which is often accompanied by insomnia, which is also more pronounced in females (p = 0.015). Females were more likely than males to report problems with nausea and vomiting (p = 0.007), which may be related to loss of appetite, which was more pronounced in females and was statistically borderline significant.

**TABLE 2.** Mean scores with standard deviations, compare differences between two independent groups with Mann-Whitney U-test for all scales of the EORTC QLQ C30 for CRC patients by gender

	Total		Male		Female		U	p
	MS	SD	MS	SD	MS	SD		
Global health status/QoL	68.8	20.1	69.8	20.1	67.4	20.3	6660.5	0.474
Physical functioning	85	18.0	86.4	17.8	83.3	18.2	6075	0.064
Role functioning	82.5	24.7	81.7	26.2	83.5	22.7	7030	0.992
Emotional functioning	86.5	17.1	89.4	14.7	82.8	19.3	5446.5	**0.002**
Cognitive functioning	90.1	15.3	92.0	11.9	87.6	18.6	6294	0.109
Social functioning	83.9	22.1	84.7	20.8	82.9	23.6	6907	0.791
Fatigue	18.1	20.3	14.3	18.5	23.1	21.4	5179	**<0.001**
Nausea/vomiting	1.3	5.4	0.4	2.5	2.4	7.5	6449.5	**0.007**
Pain	13.3	20.6	11.7	19.9	15.2	21.3	6314.5	0.122
Dyspnea	4.2	11.9	3.5	11.1	5.1	12.9	6668.5	0.286
Insomnia	20.8	28.1	16.4	24.4	26.3	31.6	5884.5	**0.015**
Appetite loss	6.1	15.9	4.2	12.5	8.6	19.1	6398	0.053
Constipation	8.2	17.9	7.0	14.8	9.8	21.1	6802	0.531
Diarrhea	8.6	17.3	8.7	16.3	8.6	18.5	6861.5	0.653
Financial problems	12.1	24.4	11.0	23.8	13.4	25.2	6520.5	0.315

1 CRC = colorectal cancer; C30 = core30; EORTC = European Organization for Research and Treatment of Cancer; MS = mean score; p = value; QoL = quality of life; QLQ = quality of life questionnaire; SD = standard deviation; U = value for U-statistics from Mann-Whitney U-tests

### EORTC QLQ CR29 scoring scale

**FIGURE 2.**
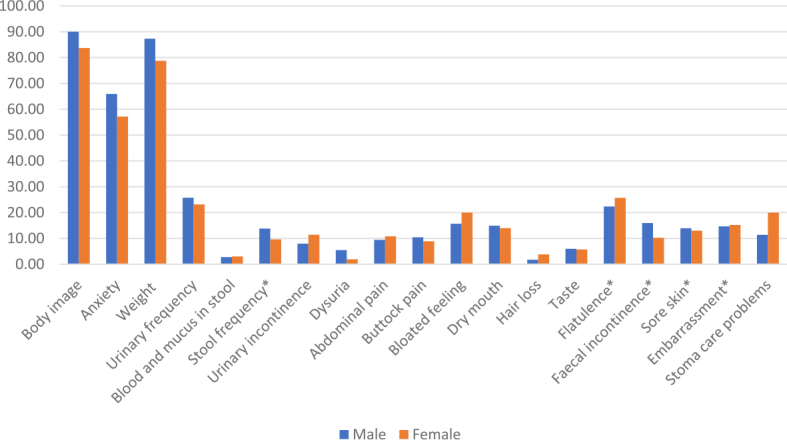
Mean scores between males and females in the EORTC QLQ CR29 questionnaire. CR29 = colorectal cancer 29; EORTC = European Organization for Research and Treatment of Cancer; QLQ = quality of life questionnaire

The results of the study show some important gender differences in the quality of life and management of symptoms and problems in CRC patients. Figure 2 presents the mean scores for the functional scales and symptoms in the CR29 in CRC patients according to gender. Males report consistently better functioning in body image, anxiety, and weight compared to females. Females report fewer symptoms in stool frequency, dysuria, buttock pain, faecal incontinence and more symptoms in urinary incontinence, bloated feeling, hair loss, flatulence, and especially stoma care problems. Results of Mann-Whitney U-tests in Table 3 presented one of the key differences is in body self-esteem, where males report better body self-esteem than females (p = 0.047). A gender difference was also evident for anxiety, where females reported more severe problems (p = 0.029). Males are less likely to report perceived weight loss or weight gain after treatment (p = 0.010), but have more problems with stool frequency (p = 0.045). They also reported more severe pain during urination (dysuria) compared to females (p = 0.008).

**TABLE 3.** Mean scores with standard deviations, compare differences between two independent groups with Mann-Whitney U-test for all scales of the EORTC QLQ CR29 for CRC patients by gender

	Total		Male		Female		U	p
	MS	SD	MS	SD	MS	SD		
Body image	87.2	21.9	90.0	18.5	83.7	25.3	6094.5	**0.047**
Anxiety	62.1	27.9	65.9	26.0	57.2	29.5	5979.5	**0.029**
Weight	83.5	25.5	87.3	22.3	78.7	28.5	5879.5	**0.010**
Sexual function (men)	35.9	26.6	35.9	26.6				
Sexual function (women)	17.2	21.9			17.2	21.9		
Urinary frequency	24.6	23.5	25.7	23.6	23.2	23.3	6598	0.394
Blood and mucus in stool	2.9	7.6	2.7	7.4	3.0	7.9	6969	0.835
Stool frequency*	12.0	17.0	13.8	17.8	9.6	15.7	6019.5	**0.045**
Urinary incontinence	9.5	20.6	8.0	20.1	11.4	21.1	6330.5	0.064
Dysuria	3.9	13.4	5.4	14.8	1.9	11.2	6311.5	**0.008**
Abdominal pain	10.0	18.1	9.4	17.6	10.8	18.8	6838	0.628
Buttock pain	9.8	19.5	10.4	20.2	8.9	18.6	6789	0.532
Bloated feeling	17.6	22.2	15.7	20.7	20.0	23.8	6407	0.179
Dry mouth	14.5	21.9	14.9	21.1	14.0	23.0	6707.5	0.463
Hair loss	2.7	10.9	1.8	9.5	3.8	12.5	6585	0.073
Taste	5.9	14.1	6.0	13.5	5.7	14.9	6859.5	0.602
Flatulence*	23.8	24.5	22.4	24.1	25.7	25.0	6528	0.293
Faecal incontinence*	13.4	22.8	15.9	24.4	10.3	20.3	6148	0.055
Sore skin*	13.5	23.0	13.9	22.9	13.0	23.3	6844	0.657
Embarrassment*	14.9	26.7	14.6	26.1	15.2	27.5	6792	0.989
Stoma care problems	14.8	26.6	11.4	22.3	20.0	31.9	418	0.321
Impotence	31.2	30.2	31.2	30.2				
Dyspareunia	10.9	24.5			10.9	24.5		

1 CRC = colorectal cancer; CR29 = colorectal cancer 29; EORTC = European Organization for Research and Treatment of Cancer; MS = mean score; p = value; QLQ = quality of life questionnaire; SD = standard deviation; U = value for U-statistics from Mann-Whitney U-tests

## Discussion

This study highlights several significant gender differences in QoL, disease-related symptoms, and late side effects among CRC survivors during the first two years after completing treatment. Our findings demonstrated that males reported better overall QoL, emotional, and social functioning than females. Emotional functioning was significantly worse in females (p = 0.002), who also experienced higher levels of fatigue (p < 0.001), insomnia (p = 0.015), and nausea/vomiting (p = 0.007). These results align with previous studies, including the EnCoRe study, which showed that poorer sleep and emotional well-being are associated with higher levels of fatigue during the first two years after CRC treatment.^20^ In the general Slovenian population, fatigue scores are also slightly higher in females than in males, although this gender difference is not statistically significant (p = 0.769). However, the rate of insomnia is higher in females than males in Slovenian population, with a difference close to statistical significance (p = 0.056).^21^ These patterns are mirrored in CRC survivors, emphasizing that fatigue and insomnia remain important problems, especially in females. In addition, body image disturbances and psychological distress have been frequently reported in CRC survivors, especially in females. In our study, we found significant gender differences in body image (p = 0.047), with females experiencing poorer body image compared to males. These findings are consistent with other studies, such as those by Reese *et al*.^22^, in which females reported lower body self-image than males, particularly in patients with rectal cancer. This could be due to the more extensive and invasive treatment, including surgery and the possibility of a stoma, which can have a greater impact on body image in females. Body image disturbances are exacerbated by changes in bowel habits, as shown in a study by Phung and Fang, in which between 25.5% and 86% of CRC survivors reported body image problems.^23^ Poor body image can lead to feelings of insecurity and reduced emotional well-being, particularly in females, who may be especially psychological vulnerable after CRC treatment. Depression and anxiety were common among CRC survivors, as found in previous studies in which anxiety rates ranged from 1.0% to 47.2%, and depression rates ranged from 1.6% to 57.0%. In terms of psychological health, our study found that females experienced more anxiety (p = 0.029) compared to males, which is consistent with other research suggesting that females are more psychologically vulnerable after CRC treatment. Higher levels of anxiety in females could also be due to socio-psychological factors, such as greater awareness of or sensitivity to emotional and physical symptoms.^24^ The change in body weight in our study could reflect physiological or hormonal gender differences in response to treatment, as well as differences in eating habits or physical activity during recovery, which should be further investigated. It could also be an important factor influencing the experience of physical selfimage and general health.

One of the unexpected findings in our study was the lack of statistically significant differences in digestive problems between males and females, although the trend suggests that males were more frequently affected by problems such as stool frequency (p = 0.045) and dysuria (p = 0.008). This is in contrasts to some other studies, where males tend to report more severe digestive symptoms after CRC treatment, particularly in relation to bowel dysfunction and sexual health. Multimodal CRC treatments, including surgery, chemotherapy and radiation, often lead to bowel dysfunction, faecal incontinence, and urinary incontinence. Although no statistically significant gender differences were seen in our study, faecal incontinence is often reported as a major problem for CRC survivors. Previous studies have shown that faecal incontinence is strongly associated with lower QoL and increased psychological distress.^25^ This discrepancy could be due to differences in sample size, patient population or the subjective nature of symptom reporting, which may vary from study to study. It would be worthwhile to investigate this further in larger, more diverse cohorts to determine if this finding holds true. These symptoms can cause significant physical and psychological distress, highlighting the need for tailored followup care.

Fatigue remains one of the most common and debilitating symptoms in CRC survivors, affecting about a third of patients after treatment.^26^ Our study confirmed that females experience significantly more frequently from fatigue than males (p < 0.001). Fatigue is a multifactorial and subjective symptom that is influenced by systemic treatments, sleep disturbances, and psychological conditions such as depression and anxiety.^27^ Similar to fatigue, insomnia was also more prevalent among females in our study. Poor sleep quality has been shown to exacerbate fatigue, as found in the EnCoRe study.^20^ Treating sleep-related problems through interventions such as cognitive-behavioral therapy or relaxation techniques could potentially improve QoL in this population.

Finally, a comparison of our results with the C30 and CR29 questionnaires reveals some interesting findings. While the C30 focuses on general QoL and functioning, the CR29 is more CRC-specific and includes symptoms directly related to bowel function and side effects of cancer treatment. In our study, significant differences were found between genders for both scales, with the C30 showing broader differences in emotional functioning, while the CR29 highlighted more specific symptoms, such as stool frequency and dysuria. This emphasises the importance of using both general and disease-specific instruments to gain a comprehensive understanding of the health and wellbeing of CRC survivors. These various symptoms and problems often persist after treatment and continue during the recovery phase. To ensure the best possible well-being and QoL, CRC survivors should be properly assess and manage for these physical and psychological symptoms.^28^

The results of this study emphasize the importance of considering gender-specific differences in CRC survivorship care. Females, in particular, may benefit from interventions targeting psychological well-being, body image and sleep quality, while males may need support for gastrointestinal and urinary symptoms. Despite the important findings, the study has some limitations. The study was based on patient self-report, which may affect the accuracy of the data collected. Males were more likely to report physical problems and less likely to report emotional problems and anxiety, which would need to be verified using a larger and more diverse sample. The lack of long-term data on quality of life has also limited the ability to assess the lasting effects of illness and treatment.

Future research should further investigate the mechanisms underlying these gender differences, including physiological, hormonal, and behavioral factors. In addition, longitudinal studies are needed to assess the long-term impact of these differences on survivorship outcomes and identify effective interventions to improve QoL for all CRC survivors. In conclusion, this study provides important insights into gender-specific differences in QoL and symptom burden among CRC survivors. Addressing these differences through personalized follow-up care may significantly improve overall well-being and QoL in this population.

## Conclusions

Our study highlights significant gender differences in quality of life and symptom burden among CRC survivors during the first two years after treatment. Females reported poorer emotional functioning, greater fatigue, insomnia, and poorer body image, while males had more frequent bowel movements and dysuria. These findings underscore the importance of gender-specific approaches in CRC survivorship care. By addressing these differences through tailored physical and psychological interventions, we can improve overall wellbeing and contribute valuable insights to research on quality of life in cancer survivorship.
